# Case Report: Treatment response of SQSTM1–ALK fusion lung adenocarcinoma to multiple ALK inhibitors

**DOI:** 10.3389/fphar.2025.1608080

**Published:** 2025-09-23

**Authors:** Jincui Gu, Jing Zhang, Shaoli Li, Ziying Lin, Lixia Huang, Yanbin Zhou

**Affiliations:** Department of Pulmonary and Critical Care Hospital, The First Affiliated Hospital of Sun Yat-sen University, Guangzhou, Guangdong, China

**Keywords:** lung adenocarcinoma, SQSTM1–anaplastic lymphoma kinase fusion, anaplastic lymphoma kinase inhibitors, case report, treatment

## Abstract

**Objectives:**

The echinoderm microtubule-associated protein-like 4 (*EML4*) gene and anaplastic lymphoma kinase (ALK) gene fusion is the most common ALK rearrangement in non-small-cell lung cancer (NSCLC). SQSTM1 (exon 5)–ALK (exon 20) fusion in patients with NSCLC was first identified in 2015. However, the treatment of lung adenocarcinoma patients with SQSTM1–ALK fusion has not been previously reported. In this study, we report for the first time an SQSTM1–ALK fusion in a stage-IV lung adenocarcinoma patient who had a variable response after sequential treatment with alectinib, ensartinib, lorlatinib, and brigatinib.

**Materials and methods:**

The biopsy specimen was subjected to hematoxylin–eosin (HE) staining, immunohistochemistry (IHC), and next-generation sequencing (NGS).

**Results:**

The patient responded to alectinib as first-line treatment and achieved stable disease for 16 months without significant toxicity. Ensartinib appeared to have a better effect on brain metastases. As third-line therapy, lorlatinib resulted in a progression free survival (PFS) of 15 months. In addition, as fourth-line therapy, brigatinib yielded a PFS of only 2 months.

**Conclusion:**

This is the first report of a patient with SQSTM1–ALK fusion who experienced different responses after treatment with alectinib, ensartinib, lorlatinib, and brigatinib. We hope that this case provides clinical evidence to guide treatment strategies for this rare variant and further supports the individualized application of ALK-tyrosine kinase inhibitors (TKIs) in patients with non-classical fusions.

## Introduction

Lung cancer is the leading cause of cancer-related deaths worldwide. Non-small-cell lung cancer (NSCLC) is currently the most common type of lung cancer, with lung adenocarcinoma being the most common histology of NSCLCs (Huber et al., 2021). However, the identification of driver mutations, including epidermal growth factor receptor (EGFR) mutations and anaplastic lymphoma kinase (ALK) rearrangements, has dramatically changed the management of NSCLC. Moreover, with the development of next-generation sequencing (NGS) technologies, the identification of new mutations is increasing the number of treatment options available to patients.

ALK rearrangement is a well-recognized oncogenic driver in NSCLC, and its rearrangement is observed in 3.4%–6.7% of NSCLC patients (Soda et al., 2007; Katayama, 2017). To date, more than 90 types of ALK fusions have been identified around the world, with the most common type being a fusion between the ALK and the echinoderm microtubule-associated protein-like 4 (*EML4*) gene (Ou et al., 2020). The first-generation ALK-tyrosine kinase inhibitor (TKI) crizotinib transformed ALK-positive NSCLC treatment, but resistance inevitably arises through ALK-dependent (secondary mutations and gene amplification) and ALK-independent (bypass pathways and small-cell transformation) mechanisms. Second-generation TKIs such as alectinib, ceritinib, ensartinib, and brigatinib were developed to address crizotinib resistance, with alectinib demonstrating 3-fold longer PFS than crizotinib in phase-III trials and becoming the first-line therapy (Kauffmann-Guerrero et al., 2021). The domestic second-generation TKI ensartinib, approved in China in November 2020, offers superior blood–brain barrier penetration, making it ideal for brain metastases (Wang et al., 2023). However, resistance to second-generation TKIs, often from ALK kinase domain mutations (e.g., G1202R and I1171N/S/T), necessitated the third-generation TKI lorlatinib. Sequential therapy with these agents is crucial for managing rare mutations and overcoming TKI resistance. Meanwhile, the rapid advances in next-generation sequencing (NGS) have enabled the detection of an increasing number of ALK fusions, such as the SQSTM1–ALK fusion. The SQSTM1–ALK fusion has been most commonly observed in large B-cell lymphomas (D'Amore et al., 2013). Iyevleva et al. (2015) identified a novel translocation partner, SQSTM1 (exon 5)–ALK (exon 20), in patients with NSCLC using NGS. However, the sequential therapy for SQSTM1–ALK fusion lung adenocarcinoma patients has not been previously reported. Only two relevant studies have been published to date. One of these studies detailed that when alectinib was used as a first-line therapy, it led to a partial response (PR) after 7 months of treatment (Rodriguez et al., 2024). The other study indicated that ensartinib managed to maintain a PR state even after 6 months of treatment (Wang et al., 2024). Notably, neither of these studies provided any information on the progression-free survival (PFS) and possible therapeutic sequences. However, for the first time, we report an SQSTM1–ALK fusion in a patient with stage-IV lung adenocarcinoma who experienced variable response after sequential treatment with alectinib, ensartinib, lorlatinib, and brigatinib. This study also marks the first reported application of lorlatinib and brigatinib in the treatment of this specific mutation site in SQSTM1–ALK fusion lung adenocarcinoma.

### Case presentation

**FIGURE 1:**
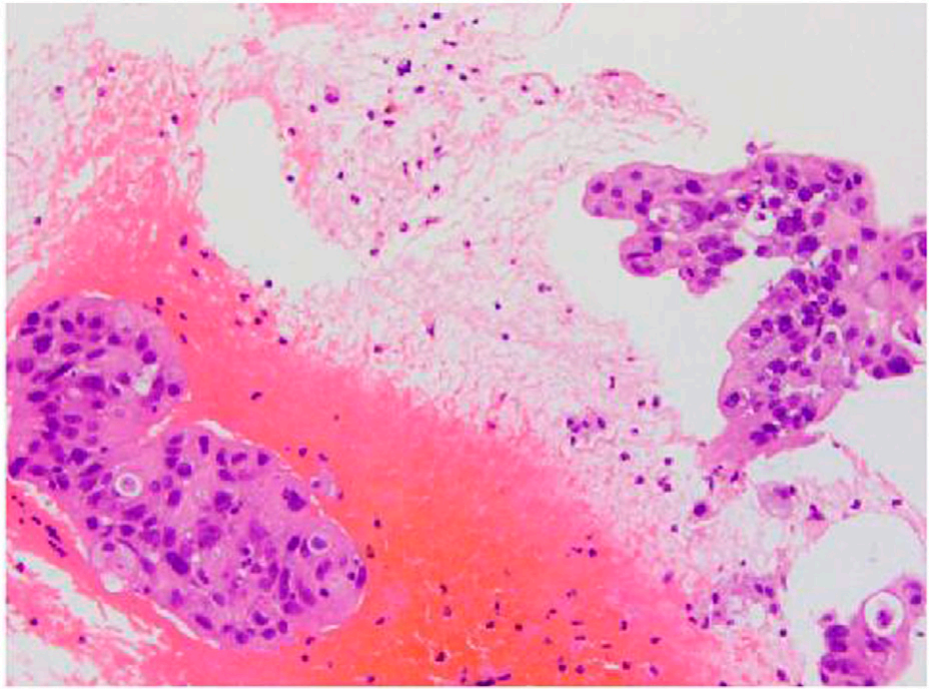
Pathologic biopsy.

**FIGURE 2:**
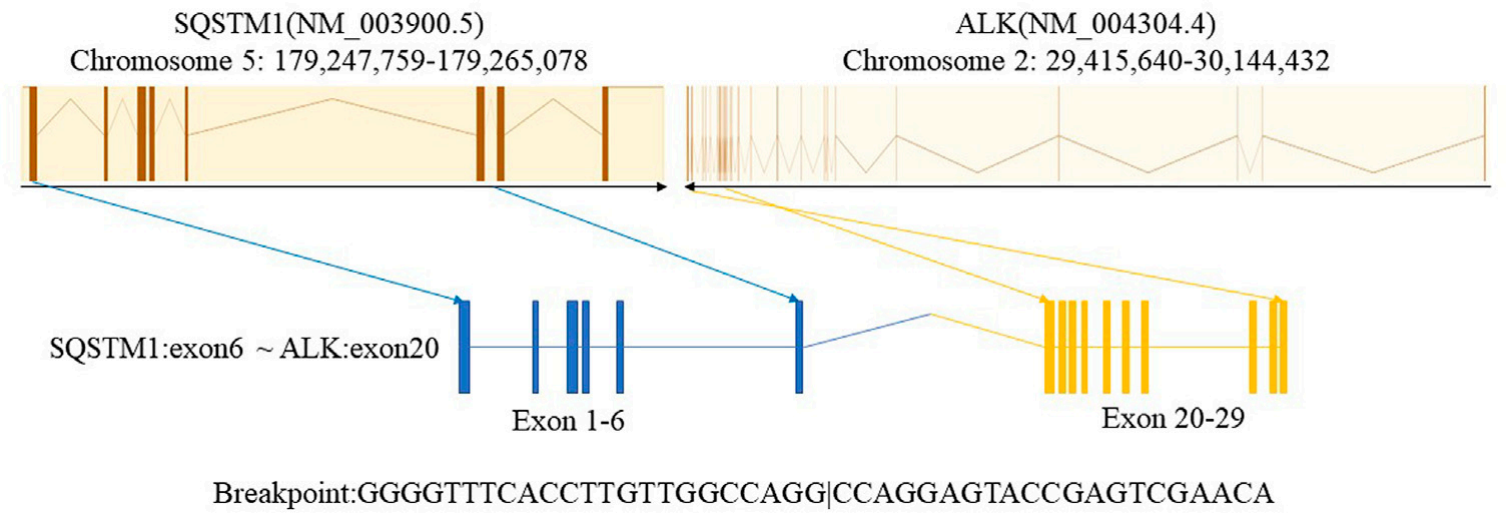
Identification of the SQSTM1–ALK fusion. ALK, anaplastic lymphoma kinase.

In January 2021, a 43-year-old man with a history of smoking presented to our hospital with chest pain and shortness of breath lasting 2 weeks. There was no relevant past medical history or family history. Diminished breath sounds in the left lung field were noted on physical examination. High-resolution computed tomography (HRCT) showed that the bronchus in the left lower lobe was narrowing with a mass of increased density and metastases of hilar and mediastinal lymph nodes, bilateral supraclavicular lymph nodes, and left pleural metastases. Emission computed tomography (ECT) demonstrated bone metastases at the first, eighth, and ninth thoracic vertebrae, which is consistent with a clinical stage of T4N3M1c (IVB). The patient’s ECOG performance status (PS) was 0. Serum tumor marker results showed elevated levels of CEA (10.79 ug/L; reference value <5 ug/L). The cytology of the pleural fluid was positive, showing a small focus of atypical cells, which was most likely lung adenocarcinoma. A lymph node biopsy was performed using endobronchial and transbronchial needle aspiration (EBUS–TBNA). Pathology revealed lymph node metastasis of lung adenocarcinoma (Figure 1). Immunohistochemical (IHC) staining was positive for TTF-1, napsin A, and CK7. P40 and CK20 were negative. Formalin-fixed paraffin-embedded (FFPE) tumor samples were analyzed by DNA next-generation sequencing (NGS, 139 gene panel, Geneseeq Technology Inc.) and RNA NGS (117-gene panel, Geneseeq Technology Inc.) The results of DNA and RNA sequencing identified an SQSTM1–ALK (exon 6-exon 20) fusion in the patient (Figure 2).

**FIGURE 3:**
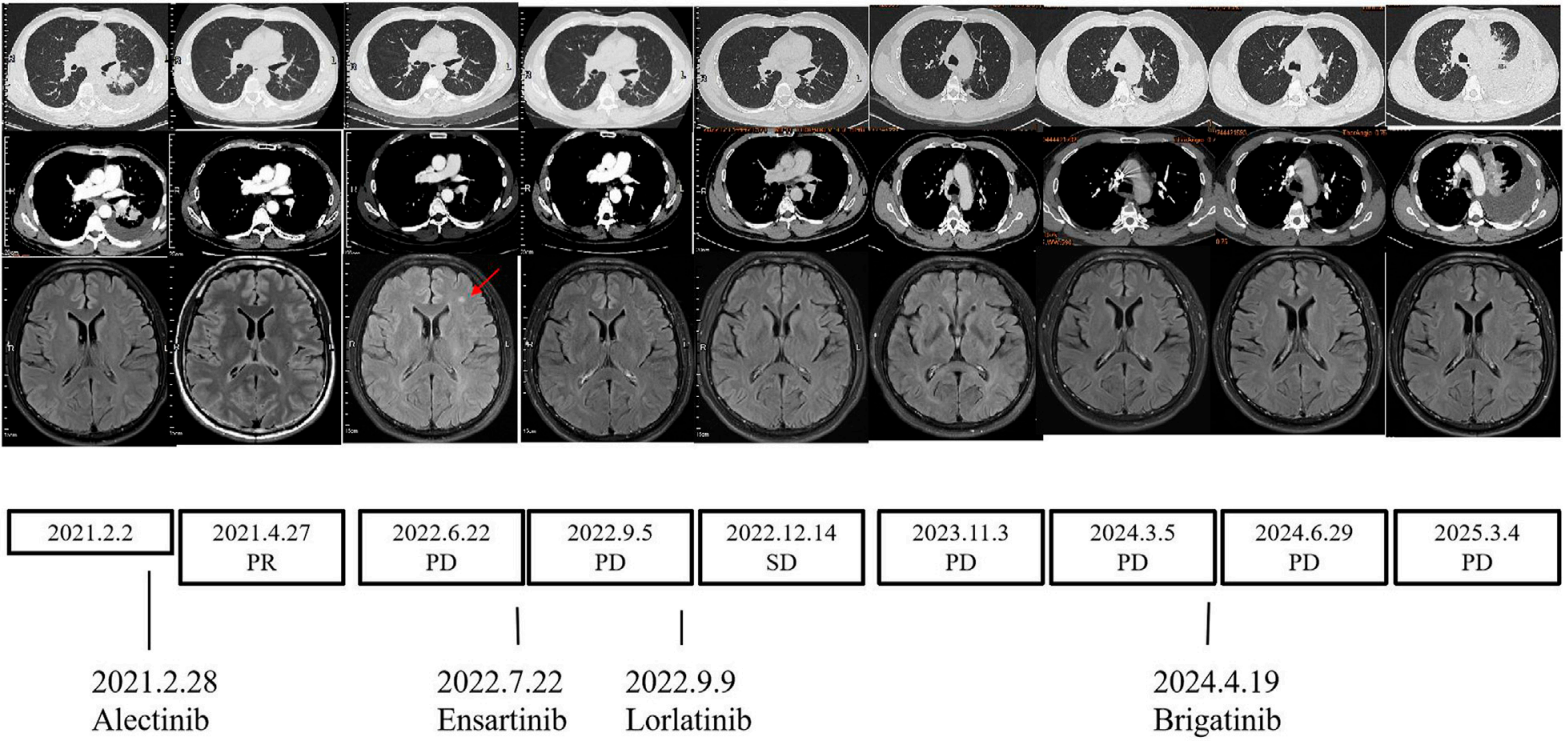
Changes during therapy. CT scans, MRI scans, and treatment timeline. CT, chest computed tomography; MRI, magnetic resonance imaging.

The patient started to receive alectinib (600 mg twice) on 28 February 2021 and bisphosphonates monthly. After 8 weeks, the CT scan demonstrated reduced tumor size and smaller lymph nodes and left pleural nodules compared with those in the previous examination, and the evaluation of therapeutic efficiency indicated partial response (PR) (Figure 3). All efficacy evaluations were performed according to the RECIST 1.1 criteria. A CT scan on 26 August 2021 showed the metastases of the hilar and mediastinal lymph nodes, and the bilateral supraclavicular lymph nodes were vanishing. Follow-up CT scans showed PR until June 2022. A CT scan on 22 June 2022 showed the thickening of the left pleural nodules, and the magnetic resonance imaging (MRI) of the brain showed one new brain metastasis of the left frontal cortex (measuring approximately 3–4 mm in diameter). The evaluation of therapeutic efficiency indicated progressive disease (PD) (Figure 3). Serum oncological markers’ results showed almost normal levels of CEA (1.37 ug/L; reference value <5 ug/L). Percutaneous transthoracic CT-guided biopsies of the left pleural lesions were negative. We conducted ctDNA analysis using DNA NGS (139-gene panel, Geneseeq Technology Inc.) with peripheral blood in July 2022, and the results still showed an SQSTM1–ALK (exon 6-exon 20) fusion in the patient. At that time, clinical evidence for sequential therapy in rare SQSTM1–ALK fusion was limited, so chemotherapy was considered an option, consistent with the NCCN guidelines (Ettinger et al., 2022), which recommend chemotherapy for TKI-progressing patients without clear targeted alternatives. We recommended re-biopsy and chemotherapy. However, the patient rejected the recommendation. Considering the higher blood–brain barrier and intracerebral distribution, the patient started to receive ensartinib (225 mg daily) on 12 July 2022. On 6 September 2022, a CT scan showed the tumor progression in the lungs; however, the MRI of the brain showed that the brain metastases had disappeared. The evaluation of therapeutic efficiency still indicated PD (Figure 3). As the patient again refused re-biopsy, treatment with lorlatinib (100 mg once daily) was initiated on 9 September 2022. Only grade-1 hypercholesterolemia and liver function impairment occurred during lorlatinib treatment. Until November 2023, during the period of lorlatinib treatment, the lesions showed stable conditions. In November 2023, upon detecting disease progression (manifested by significant enlargement of the pleural nodule in the dorsal segment of the left lower lobe), repeat pathological biopsy and genetic testing were conducted. The results confirmed that the patient still had SQSTM1–ALK lung adenocarcinoma, and PD-L1 expression testing was also performed and was positive (TPS = 40%). Following a comprehensive evaluation by our medical team, we recommended a change in the treatment regimen. However, in the absence of obvious symptoms, the patient requested to continue the current medication and independently sought traditional Chinese medicine treatment. However, disease progression was confirmed by April 2024 CT findings, which showed significant enlargement of the pleural nodule in the dorsal segment of the left lower lobe. In addition, the treatment was switched to brigatinib (180 mg once daily). However, disease progression was re-detected in June 2024, manifested by the enlargement of the pleural nodule in the left lower lobe and the concurrent enlargement of other nodules in the left oblique fissure and pleura. Consistent with previous instances, after a comprehensive evaluation, our team recommended that the patient switch to a different treatment regimen. However, as the patient was unwilling to undergo chemotherapy, they insisted on continuing brigatinib treatment and additionally started taking traditional Chinese medicine. Nevertheless, the disease was still advancing when the patient was evaluated in March 2025. Moreover, the patient experienced severe shortness of breath. At this point, the treatment was changed to a combination of chemotherapy and antiangiogenic therapy.

Initially, the patient felt hopeful during alectinib treatment as the disease stabilized, but anxiety grew with tumor progression. Ensartinib relieved concerns about brain metastases, and lorlatinib brought another period of stability. However, brigatinib’s short-lived efficacy was disappointing. Throughout the treatment, the patient remained determined to fight, yet the continuous treatment changes and disease progressions took an emotional toll, leaving him hoping for more effective treatments.

## Discussion

To our knowledge, this is the first reported case of a lung adenocarcinoma patient with a rare ALK rearrangement (SQSTM1–ALK) who was successfully treated with alectinib, ensartinib, lorlatinib, and brigatinib sequentially. In this study, we report the novel therapeutic application of lorlatinib and brigatinib in a patient with SQSTM1–ALK fusion-positive lung adenocarcinoma harboring this unique mutation. Even as the third-line therapy, lorlatinib has a PFS of 15 months.

The *SQSTM1* gene, located on 5q35, encodes a ubiquitin-binding protein that mediates the NF-kB pathway activation and participates in oxidative stress response and autophagy (Komatsu et al., 2012; Pankiv et al., 2007). Previously, mutations of SQSTM1 were reported in Paget bone disease (Laurin et al., 2002). Iyevleva et al. (2015) used NGS to identify a novel translocation partner, SQSTM1 (exon 5)–ALK (exon 20), in patients with NSCLC. Honda et al. (2019) reported a patient with an inflammatory myofibroblastic tumor harboring an SQSTM1–ALK fusion who exhibited a marked response to alectinib treatment, which was sustained for 17 months following systemic therapy initiation without significant adverse events. To date, only two studies have reported treatment outcomes for SQSTM1–ALK fusion-positive lung adenocarcinoma: one demonstrated a durable partial response (PR) after 7 months of first-line alectinib therapy (D'Amore et al., 2013), while another showed sustained PR for 6 months with ensartinib therapy (Iyevleva et al., 2015). Clinical evidence for SQSTM1–ALK fusion-positive lung adenocarcinoma remains limited, especially for lorlatinib and brigatinib.

Due to the lack of clinical evidence for targeted drug use in this rare mutation, we also referred to the targeted drug selection strategies for other rare ALK mutations and common ALK mutations when formulating the treatment regimen. In most cases of uncommon ALK fusions (Lin et al., 2022; Li et al., 2024; Liu et al., 2023), alectinib is preferred and has shown favorable efficacy. In addition (Li et al., 2021), in cases with the novel LMO7–ALK fusion, alectinib was administered first with a PFS of 2 months, followed by ensartinib, which achieved a PFS of 18 months. The treatment regimen for our patient with a rare SQSTM1–ALK fusion is similar to those used for other patients with rare ALK mutations mentioned above. In addition, for common ALK fusion-positive lung adenocarcinoma patients, second-generation ALK-TKIs such as alectinib were approved as first-line treatments due to their superior systemic and CNS efficacy and favorable safety compared to chemotherapy and crizotinib (Novello et al., 2018). The ALEX study showed that alectinib outperformed crizotinib in PFS regardless of EML4–ALK variants (Camidge et al., 2019). Gainor et al. (2016), Noe et al. (2020) found that the most common mutations following alectinib treatment failure are G1202R and I1171N/S/T. In addition, mutations at the L1198, V1180, I1171N, and L1196 sites affect conformational changes in the ATP-binding pocket and hinge region, thereby altering the “latching” interaction between alectinib and ALK. For instance, two “gatekeeper” mutations (V1180L and ALK L1196M/Q) and I1171T/N/S mutations confer resistance to both alectinib and crizotinib but remain sensitive to later-generation inhibitors such as ceritinib, brigatinib, and lorlatinib (Latif and Liu, 2020) (Makuuchi et al., 2018; Okada et al., 2019). Another second-generation TKI, ensartinib, is 10-fold more potent than crizotinib in inhibiting the growth of ALK-positive lung cancer cells. The eXalt3 trial demonstrated its superiority over crizotinib in PFS and its intracranial prophylactic role (Horn et al., 2018) (Horn et al., 2021). In our patient, ensartinib also showed favorable therapeutic effects on brain lesions. However, studies have shown that the most common secondary resistance mutations in patients with disease progression after ensartinib treatment are G1269A, G1202R, and E1210K. Among these, G1269A remains sensitive to other second-generation inhibitors, while G1202R demonstrates the most definite response to lorlatinib (Horn et al., 2019; Yang et al., 2021; Yang et al., 2020). Lorlatinib, a third-generation ALK-TKI, was developed to overcome resistance to earlier-generation TKIs (Johnson et al., 2014; Shaw et al., 2019a) and achieve marked central nervous system (CNS) penetration. A global phase-II study showed an overall response rate (ORR) of 47% and an intracranial response rate of 63% in patients pre-treated with at least one ALK-TKI (Solomon et al., 2018) (Shaw et al., 2019b). Brigatinib is a second-generation ALK-TKI. The ALTA trial series confirmed its efficacy (Yoshida et al., 2023), and intriguingly, it demonstrated *in vitro* inhibitory activity against 17 ALK mutants associated with resistance to crizotinib, ceritinib, and alectinib. Specifically, brigatinib showed more than 3-fold greater potency than alectinib against L1152R, I1171N, V1180L, L1196M, G1202R, and G1269A mutations, and it even exhibited superior activity compared to lorlatinib against the G1269A mutation (Horn et al., 2019). After lorlatinib progression, some compound mutations (e.g., V1185L + L1196M) can re-sensitize to first-/second-generation TKIs. Additionally, brigatinib achieves a 7-month mPFS even in patients previously treated with ceritinib, alectinib, or at least two ALK-TKIs (Popat et al., 2021; Descourt et al., 2019; Nishio et al., 2021). Moreover, most studies have reported the SQSTM1 (exon 5)–ALK (exon 20) fusion. In contrast, our case’s SQSTM1 (exon 6)–ALK (exon 20) fusion, with the breakpoint in SQSTM1 exon 6 (closer to the coding region), may alter the fusion protein’s conformation and impair ALK-TKI binding. Such variants may show reduced sensitivity to drugs such as brigatinib due to steric hindrance from the fusion site’s proximity to the ALK kinase domain (Kauffmann-Guerrero et al., 2021). Unfortunately, it did not yield a favorable response. Since no tissue genetic testing was performed after lorlatinib resistance, it cannot be ruled out that, first, there was ALK-independent bypass resistance; second, brigatinib did not cover the resistance sites specific to ALK itself. Due to the patient’s refusal to undergo a third genetic test, chemotherapy was ultimately initiated.

STAT3 is a key regulator of ALK-induced growth and survival (Chiarle et al., 2005; Zamo et al., 2002; Amin et al., 2004). SQSTM1–ALK expression is associated with high STAT3 phosphorylation at Tyr705, which is crucial for its dimerization and DNA binding (D'Amore et al., 2013). Alectinib strongly inhibits ALK and its downstream signaling molecules, including STAT3 (Yoshimura et al., 2016). Moreover, ALK-rearranged lung cancer cell survival after ALK inhibition is mainly dependent on the STAT3 activity (Yanagimura et al., 2022). This is probably one of the reasons why lung adenocarcinoma patients with SQSTM1–ALK fusion are successfully being treated with alectinib, ensartinib, and lorlatinib. In addition, we speculate that the differential efficacy of different drugs may be related to the inhibition efficiency of ALK phosphorylation and the blood–brain barrier permeability.

## Conclusion

This is the first report on a patient with SQSTM1–ALK fusion who had a different response after sequential treatment with alectinib, ensartinib, lorlatinib, and brigatinib. This case is the first to report the response data of SQSTM1 (exon6-ALK exon20) fusion to ensartinib and alectinib, providing clinical evidence for the treatment strategy of this rare variant and further supporting the individualized application of ALK-TKIs in patients with non-classical fusions.

## Data Availability

The original contributions presented in the study are included in the article/supplementary material, further inquiries can be directed to the corresponding authors.
